# Bimekizumab: Short-Term Effectiveness and Safety in Real Clinical Practice in Andalucia, Spain

**DOI:** 10.3390/life14030281

**Published:** 2024-02-20

**Authors:** Ricardo Ruiz-Villaverde, Lourdes Rodriguez-Fernandez-Freire, Marta Cebolla-Verdugo, Alvaro Prados-Carmona, Carlos Hernández-Montoya, José Carlos Armario-Hita, Manuel Galán-Gutiérrez

**Affiliations:** 1 Hospital Universitario San Cecilio, 18016 Granada, Spain; martacevers@gmail.com (M.C.-V.);; 2 Instituto Biosanitario de Granada (ibs.GRANADA), 18014 Granada, Spain; 3 Hospital Universitario Virgen del Rocio, 41013 Sevilla, Spain; lourdesrff@gmail.com; 4 Hospital de Poniente, 04700 Almeria, Spain; carlhemo@gmail.com; 5 Hospital Universitario de Puerto Real, 11510 Cádiz, Spain; jcarmarioh@gmail.com; 6 Hospital Universitario Reina Sofía, 14004 Córdoba, Spain

**Keywords:** psoriasis, bimekizumab, biological therapy

## Abstract

**Introduction**: Psoriasis, a chronic inflammatory skin disease, affects 2–10% of the population globally. Bimekizumab (BMK), a monoclonal antibody targeting IL-17, is a dual inhibitor of IL17 A and F that has shown efficacy in treating moderate to severe plaque psoriasis. This real-world evidence (RWE) study aims to assess BMK’s efficiency and safety in naïve and refractory patients. **Material and methods:** A retrospective analysis of a multicenter observational study included 22 patients treated with BMK from April 2023 to February 2023 in five Andalusian hospitals. Ethical approval was obtained, and patients provided informed consent. Assessment criteria encompassed Psoriasis Area and Severity Index (PASI), body surface area (BSA), VAS pruritus, Dermatology Life Quality Index (DLQI), and minimum disease activity (MDA) at 0, 4, 12, and 24 weeks. **Results:** Patients, predominantly with plaque psoriasis, exhibited significant improvements in PASI (baseline 15.7 to 0.4 at week 16), BSA (baseline 20.7 to 0.43 at week 16), DLQI (baseline 17.93 to 0.43 at week 16), and pruritus (baseline 7.12 to 0.4 at week 16). At week 16, 95.4% achieved MDA. No safety concerns or treatment discontinuations were reported. **Discussion:** This RWE study aligns with pivotal clinical trials, confirming BMK’s efficacy and safety. Notably, BMK demonstrated rapid and sustained psoriasis clearance, even in challenging areas. The study’s limitations include a small sample size, suggesting the need for further exploration of patient-reported outcomes. **Conclusion:** Bimekizumab exhibited optimal efficacy and safety profiles in treating moderate to severe plaque psoriasis in a real-world setting. Rapid response, sustained clearance, and favorable safety outcomes contribute to improved patient experiences. Future research could delve into patient-reported outcomes and expand sample sizes to enhance the understanding of BMK’s real-world effectiveness.

## 1. Introduction

Psoriasis is a chronic and recurrent inflammatory skin disease of genetic basis, immunologically mediated, that affects almost 2.7% of the population in Spain [1]. Plaque or vulgar psoriasis is the most common form and represents around 85–90% of cases. It is characterized by the presence of raised erythematous-scaly plaques, well defined, generally distributed symmetrically in areas of extension of the extremities, scalp and, to a lesser extent, at the palmo-plantar level. Inflammatory arthropathy (psoriatic arthritis) develops in up to 30% of cases [2,3].

It can be triggered and/or aggravated by multiple factors. The prevalence of psoriasis varies in the European Union between 2% and 6% [4,5], being similar in both genders [6]. It should be considered a multifactorial disease, whose etiology is still unclear, although it is mediated by the immune system [6], characterized by the hyperproliferation of keratinocytes and skin-infiltrating T lymphocytes that overexpress proinflammatory mediators [4]. On the other hand, the existence of a genetic predisposition (approximately 40% have a family history of psoriasis), as well as certain related environmental triggers [7], such as stress, smoking, obesity, alcohol consumption, viral or bacterial infections, vitamin D deficiency, and some drugs, seem to intervene in its development.

The main goal of psoriasis treatments is to achieve and maintain the broadest possible clearance of the lesions in the long term [8], as well as the control of long-term systemic inflammation and the prevention of the appearance and progression of systemic comorbidities.

Treatment will depend on the severity of the disease, which will be determined, among other things, by the extent of the disease, the location of the lesions, and the degree of inflammation, as well as the impact on quality of life [9,10]. In those cases characterized by mild or restricted psoriasis, topical therapies like glucocorticoids, vitamin D derivatives, or a synergistic blend thereof are employed for management. If psoriasis cannot be controlled with topical treatments, involves areas of functional compromise, or is associated with psoriatic arthritis, systemic therapies may also be required. Treatment options include phototherapy (ultraviolet A, ultraviolet B, or narrowband ultraviolet B radiation), photochemotherapy (such as psoralens plus ultraviolet A radiation), acitretin, systemic immunosuppressive agents (cyclosporine, methotrexate, and apremilast), fumaric acid esters or biological agents targeting tumor necrosis factor-alpha (TNF-α) (adalimumab, etanercept, infliximab, certolizumab), interleukins IL-12 and 23 (ustekinumab), IL-17 (secukinumab, brodalumab, ixekizumab, and bimekizumab), or IL-23 (guselkumab, tildrakizumab, and risankizumab).

Bimekizumab (BKZ) is a humanized IgG1/κ monoclonal antibody with two identical antigen-binding regions that selectively binds with high affinity to the cytokines IL-17A, IL-17F, and IL-17AF, and, thus, blocks their interaction with the IL-17RA/IL-17RC receptor complex.

The interleukin-17 (IL-17) pathway plays a pivotal role in the pathogenesis of psoriasis. IL-17, primarily produced by T helper 17 (Th17) cells, acts as a key mediator in the immune response and inflammatory processes. In psoriasis, there is an overactivation of Th17 cells and an upregulation of IL-17 expression, leading to the recruitment of immune cells and the release of proinflammatory cytokines, chemokines, and growth factors.

The IL-17 cytokine family comprises six isoforms (IL-17A to IL-17F), with IL-17A being the most extensively studied in the context of psoriasis. The binding of IL-17A to its receptor (IL-17RA) triggers a cascade of intracellular signaling pathways, including the activation of NF-κB, MAPK, and C/EBP, which collectively contribute to the inflammatory response observed in psoriatic lesions. This sustained inflammation results in abnormal keratinocyte proliferation, angiogenesis, and immune cell infiltration, leading to the characteristic features of psoriasis.

Interleukin-17F (IL-17F) is another member of the IL-17 cytokine family that, like IL-17A, has been implicated in the pathogenesis of psoriasis. The overexpression of IL-17F, mainly derived from activated T helper 17 (Th17) cells, contributes to the sustained inflammation and aberrant immune response observed in psoriatic lesions. IL-17F shares structural and functional similarities with IL-17A, and both cytokines can act synergistically to amplify inflammatory cascades. Blocking the IL-17F pathway has emerged as a promising therapeutic strategy in psoriasis management.

Bimekizumab inhibits these proinflammatory cytokines, resulting in an improvement in skin inflammation and, thus, improvement in psoriasis-related clinical symptoms.

The recommended dose of bimekizumab is 320 mg (given as two subcutaneous injections of 160 mg each) at weeks 0, 4, 8, 12, and 16 and every 8 weeks thereafter. Injections must be administered in different anatomical locations.

Discontinuation of treatment should be considered in patients who have not shown improvement after 16 weeks.

The authorization of bimekizumab is based on the results of three multicenter, randomized, double-blind, placebo- or active-comparator-controlled phase III clinical trials. They evaluated the efficacy and safety of bimekizumab compared to adalimumab in the PS0008 study (BE SURE), compared to placebo and ustekinumab in the PS0009 study (BE VIVID), and compared to placebo in the PS0013 study (BE READY) [11,12,13].

Patients included in the BE SURE, BE VIVID, and BE READY studies were ≥18 years of age with moderate to severe chronic psoriasis (with or without psoriatic arthritis), with a Psoriasis Severity and Extent Index (PASI) score and Severity Index ≥ 12 and a body surface area affected by psoriasis (BSA) ≥ 10%, an Investigators Global Assessment (IGA) score ≥ 3 on a 5-point scale, and were candidates to receive systemic treatment and/or phototherapy for psoriasis and candidates for treatment with adalimumab (BE SURE study) or ustekinumab (BE VIVID study).

This is a real-world evidence (RWE) study that evaluates the efficiency and safety of BMK on the short and mid-term in our daily clinical practice.

## 2. Objective

To evaluate the efficiency and safety of BMK in naïve patients and refractory patients who failed or present an inadequate response to previous biological therapy: anti-TNFα, anti-IL17, anti-IL12/23, or anti IL23.

## 3. Material and Methods

### 3.1. Study Design

This constitutes a retrospective examination of a multicenter, observational study delineating actual clinical practices, encompassing individuals undergoing treatment for moderate-to-severe plaque psoriasis (PSO) with BMK. The cross-sectional analysis encompasses data from patients recorded between April 2023 and February 2023. The study enlisted the collaboration of five tertiary hospitals in Andalusia, Spain. Ethical approval for this study was obtained from the Ethics Committee of Hospital Universitario San Cecilio (DER-HUSC-006_2023). Prior to inclusion in the study, patients provided informed consent.

### 3.2. Patients

Inclusion criteria were (1) adult moderate-to-severe plaque PSO patients; (2) PSO diagnosis since ≥6 months; (3) patients who experimented unspecified inefficacy, primary or secondary failure to anti-TNFα, anti-IL17, anti-IL12/23, anti IL23, as well as patients who discontinued treatment due to adverse events (AE) or patients naïve to any of the previously mentioned biological treatments; (4) patients who had received at least one injection of BMK according to the data in the technical data sheet and according to prescription in routine clinical practice.

Exclusion criterion was patients who did not give informed consent to participate in the study.

### 3.3. Treatment

Patients received BMK following data sheet specifications (the recommended dose of bimekizumab is 320 mg (given as two subcutaneous injections of 160 mg each) at weeks 0, 4, 8, 12, and 16 and every 8 weeks thereafter).

### 3.4. Outcome Measures

Disease severity and treatment response was assessed by absolute Psoriasis Area and Severity Index (PASI), body surface area (BSA), VAS pruritus, Dermatology Life Quality Index (DLQI), and minimum disease activity (MDA) at 0, 4, and 16 weeks. Any reason for discontinuation was reported and used for the analysis. Continuous variables are presented as mean ± standard deviation, and discrete variables are presented as relative and absolute frequencies.

Measurements of disease involvement can be objective (Psoriasis Activity Skin Index, PASI; the body surface area, or percentage of affected skin, BSA; the Physician Global Assessment PGA), or subjective, performed by the patient (Dermatology Life Quality Index, DLQI); although all have intrinsic limitations to each index.

MDA includes the dermatologist’s assessment of BSA, the presence or absence of a special location, and the exclusion of arthritis. The patient scores the DLQI and quantifies the intensity of itching (0–10), erythema (0–10), peeling (0–10), and visibility (0–10). MDA is met when there is no active arthritis, plus three of the following items: itch ≤ 1/10; peeling ≤ 2/10; redness ≤ 2/10; visibility ≤ 2/10; BSA ≤ 2; DLQI ≤ 2, and there are no lesions in special locations.

Primary failure was considered failure to reach PASI < 3 after applying the biological treatment, and secondary failure was defined as failure to maintain PASI < 3 after 12 weeks of treatment. No laboratory testing was assessed.

### 3.5. Safety

Safety and tolerability to BMK were evaluated during the follow-up of the study (any adverse event experienced by the patient was reported). 

### 3.6. Statistical Analysis

No data imputation was performed. Data are presented as mean ± standard deviation (SD) for continuous variables, and number and percentage for categorical variables. A *p* < 0.05 was considered statistically significant. All analyses were performed using GraphPad Prism version 8.0.0 for Windows (GraphPad Software, San Diego, CA, USA, www.graphpad.com, accessed on 15 November 2023).

## 4. Results

A total of 22 patients (10 men and 12 women) were included, with a minimum follow-up of 16 weeks. In our study, the mean age was 44 years (range 25–64). All patients had plaque psoriasis (PsO) as their main clinical diagnosis (Table 1). Regarding difficult-to-treat areas, 45.5% (*n* = 10) had nail involvement, 72.7% (*n* = 16) had scalp involvement, 13.6% (*n* = 3) had palmo-plantar involvement, and 54.5% (*n* = 12) had inverse psoriasis, with 41% (*n* = 9) having genital psoriasis. Three of them had psoriatic arthropathy (13.63%). Our patients were overweight (41% were overweight and 41% had some degree of obesity). Concerning psoriatic and nonpsoriatic comorbidities, the most frequent referred was arterial hypertension (27.3%). A total of 22.7% of the patients in our study had dyslipidemia and depression as the main psychological comorbidity. Three patients also had latent tuberculosis infection that had been previously treated with Isoniazid 300 mg/d for 9 months, according to the protocols of the hospitals that participated in the present study.

Regarding the therapeutic history of our patients, three patients were naïve to biologic therapy. The remaining patients had undergone treatment with at least one biologic drug to control their moderately severe psoriasis, the distribution being as follows: eight patients had used one biologic therapy, two patients had two treatments, four patients had three previous therapies, three patients had five treatments, one patient had six therapies, and one patient had used eight biologic drugs previously. All showed great improvement in their condition, with rapid achievement of the established primary endpoints. 

**Figure 1:**
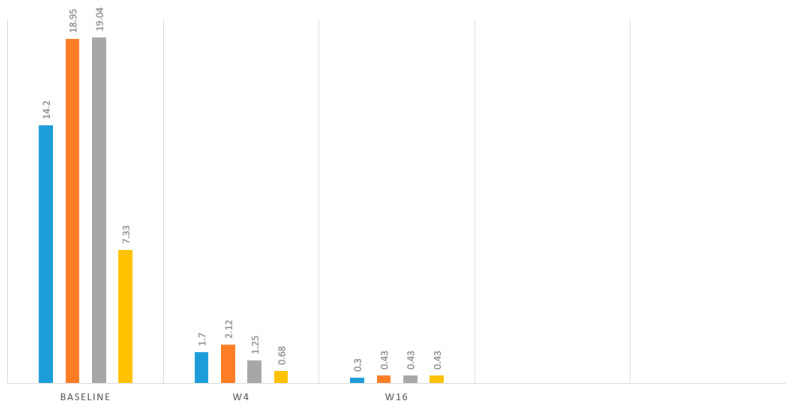
Evolution of mean PASI, BSA, DLQ, and AVS pruritus values. Blue: PASI, Orange: BSA, Grey: DLQI, Yellow: VAS pruritus.

**Figure 2:**
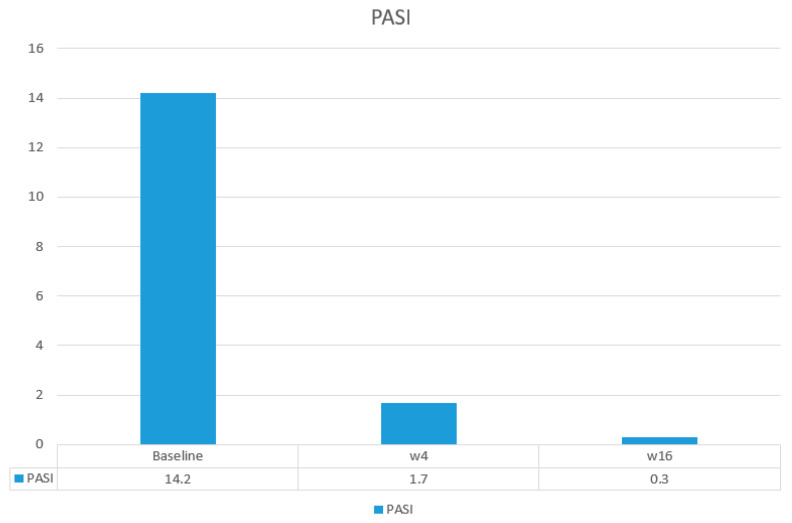
Evolution of PASI in the short term.

**Figure 3:**
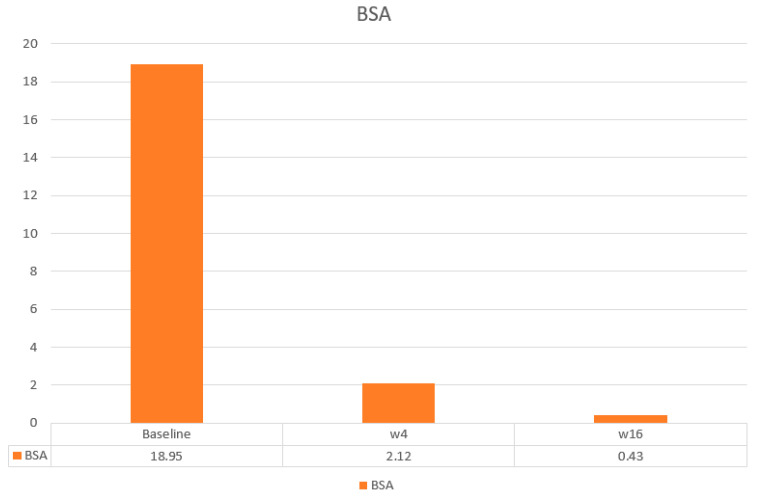
Evolution of BSA in the short term.

**Figure 4:**
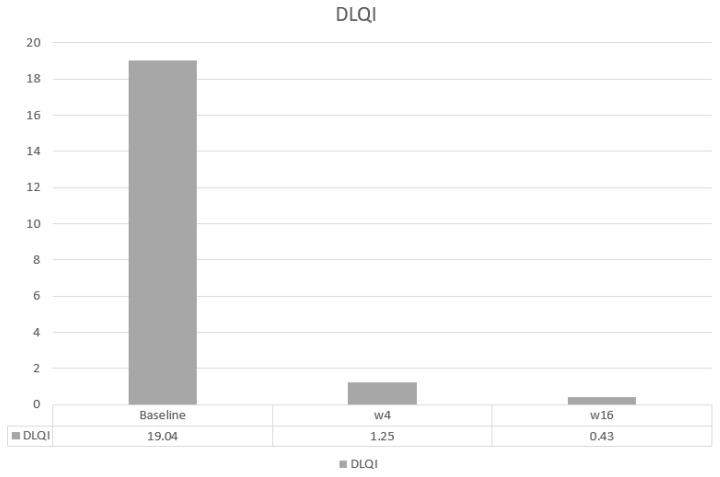
Evolution of DLQI in the short term.

**Figure 5:**
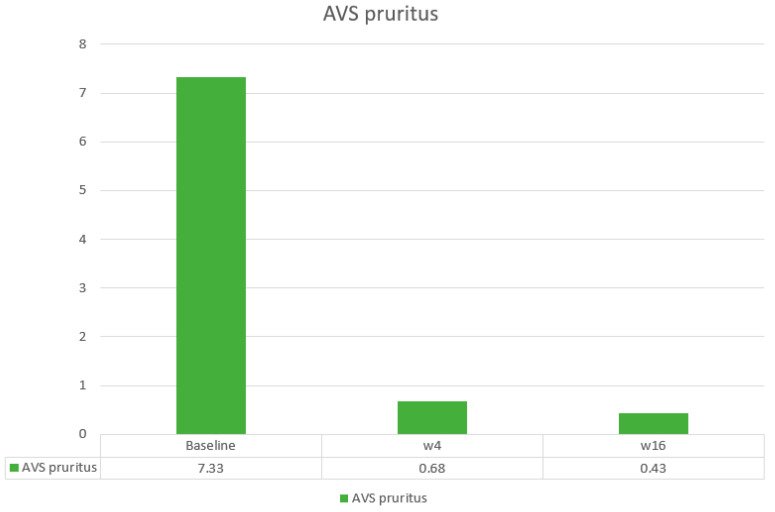
Evolution of Analogical Visual Scale of pruritus in the short term.

The main efficacy measures assessed showed the following results: Mean baseline PASI went from 15.7 to 1.7 at week 4 and to 0.4 at week 16. Body surface area affected (BSA) went from a mean baseline value of 20.7 to 2.12 at 4 weeks and 0.43 at week 16. DLQI values showed a mean baseline value of 17.93, 1.25 at week 4, and 0.43 at 16 weeks. The visual analogue pruritus scale went from 7.12 at baseline to 0.68 at week 4 and 0.4 at week 16. We present the evolution of each efficiency variable in a segregated way in the different graphs (Figure 1, Figure 2, Figure 3, Figure 4 and Figure 5).

**Figure 6:**
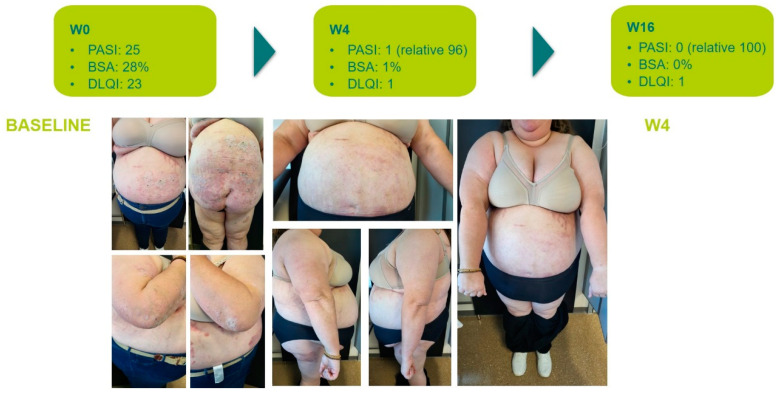
Clinical evolution after 4 weeks of the patient with the highest BMI in the series (naïve to biologics).

Specifically, we assessed whether minimal disease activity (MAD) was achieved in our patients. At week 4, 15 of our 22 patients (68%) had achieved MAD. At week 16, all but 1 patient (95.4%) had achieved the parameters to consider that the patient met all the characteristics of minimal disease activity achieved (Figure 6)

In addition, we analyzed the therapeutic response in difficult-to-treat areas (scalp, nails, palmo-plantar, and genital/inverse). Only three patients had plaque psoriasis without involvement of any of these sites. Of the 19 patients with one or more of these lesions, all showed complete improvement of the lesions except for 1 patient with scalp, nail, and genital involvement, who showed improvement but did not achieve complete clearance in the genital and nail areas.

In terms of safety, no analytical alterations (neutropenia), upper respiratory tract infections, candidiasis, or any other adverse event that could lead to temporary or definitive discontinuation of treatment were reported.

## 5. Discussion

The assessment of the results obtained in real clinical practice are based on the results of the pivotal clinical trials that led to the authorization of BMK. The efficacy of BMK is based on the results of three multicenter, randomized, double-blind, placebo- or active comparator-controlled Phase III clinical trials. These clinical trials have evaluated the efficacy and safety of bimekizumab compared to adalimumab in study PS0008 (BE SURE), compared to placebo and ustekinumab in study PS0009 (BE VIVID) and compared to placebo in study PS0013 (BE READY).

Baseline characteristics were consistent across the three studies. Participants were predominantly male (70.7%) and white (84.1%), with a mean age of 45.2 years (18–83 years), and 8.9% were ≥65 years. In our study, however, the proportion of female patients was higher and this is probably due to the bias in many clinical trials to enroll women because of the contraceptive measures required.

The median baseline BSA was 20%, the median baseline PASI score was 18, and the baseline IGA score was severe in 33% of patients. The median baseline PSD pain, itching, and scaling scores were between 6 and 7 on a 0–10 point scale and the median baseline DLQI total score was 9. These data are perfectly comparable to those presented in our study.

A total of 38% of patients had received prior biologic therapy; 23% had received at least one anti-IL17 drug (primary anti-IL17 failures were excluded), and 13% had received at least one anti-TNF drug. Twenty-two per cent had not received any previous systemic treatment (including biologics and nonbiologics). In our area and due to existing regulatory measures, it is difficult for patients to start innovative biologic therapy after systemic treatment. It is relevant in most cases to start with biosimilar treatment [14].

To date, we have found only one publication of results in actual clinical practice, the results of which correspond to the Italian registry of advanced therapies for the management of moderate–severe psoriasis: IL PSO (Italian landscape psoriasis) [15]. In relation to baseline data, the mean age of the patients was 48.86 years, and 68.4% were males. These data are in line with the baseline data from the previously discussed pivotal clinical trials. The mean body mass index (BMI) was 26.93 kg/m^2^ (SD 5.21). In this case, we were surprised by these data as up to 25% of patients are classified as obese. On the other hand, more than half of patients (50.6%) had at least one cardiometabolic comorbidity, including obesity, arterial hypertension, cardiovascular disease, type II diabetes mellitus, and hypercholesterolemia. Twenty-five patients (10.6%) had a concomitant diagnosis of psoriatic arthritis, and data were in agreement with our series.

One hundred and thirty-four patients were naïve to biological therapies (56.5%). As previously mentioned, these data, which are more in line with the usual clinical trials, differ from our clinical practice, since in our case, only 13.6% of patients were naïve to biologics. 

This may have repercussions on the efficacy results, since in almost all clinical practice series presented to date for the different biologic drugs, but mainly anti-IL23 and anti-IL17, the response is initially better when the patient has not failed successively to different drugs for the treatment of their moderate–severe psoriasis [16,17]. 

One hundred and forty-six patients (61.6%) had the involvement of at least one difficult-to-treat area (scalp/face, palms/soles, genital, or nails). Severe impairment of the quality of life (with a DLQI ≥ 10) was reported by 194 patients (81.9%).

One of the main characteristics of BMK regards its rapid response, and this is perfectly verifiable in our series. This issue is shared with the clinical trials in terms of relative efficacy (PASI90, PASI100) [11,12,13] in the Italian series (data also expressed in terms of relative efficacy) [15] and in our series where, on average, we reached an average PASI of less than 2 at week 4, which is within the therapeutic objectives postulated by the Spanish Psoriasis Group in the latest update of their guidelines [18,19].

One of the main novelties that our series presents is the determination of the MAD. This concept has been widely developed in its measurement in different rheumatological conditions but not in psoriasis [20]. To date it has only been used on one occasion to determine the effectiveness of switching from ustekinumab to guselkumab [21]. In our series, it is clearly observed how the administration of BMK achieves that almost all patients enter MDA (68% at w4 and 95.4% at w16), which can result in therapeutic optimization techniques given the difficulty of prescribing in our clinical practice. Pharmacoeconomic studies are necessary to show us the correlation between the achievement of the MAD and the cost that can be derived from the optimization or lengthening of the therapeutic interval if the previously acquired levels of efficacy are maintained.

Concerning safety, the most commonly documented adverse events (AEs) across various pivotal clinical trials (with a prevalence at least 1% greater than the placebo group) included infections (20.4% BKZ vs. 6.6% placebo), predominantly nasopharyngitis (9.4%) and oral candidiasis (7.6%). Additionally, pharyngitis, folliculitis, tinea pedis, oropharyngeal candidiasis, and headache were reported at a frequency of ≥1% higher than in the placebo group. Over an extended duration, the most frequently reported AEs associated with BKZ encompassed infections, skin and subcutaneous tissue disorders, and gastrointestinal disorders [11,12,13]. Opportunistic infections were mainly localized fungal events. Most infections consisted of mild or moderate upper respiratory tract infections. There were higher rates of oral and oropharyngeal candidiasis in patients treated with bimekizumab. More than 98% of the cases were nonsevere, of mild or moderate intensity, and did not require treatment suspension.

In Gargiulo’s series [15], no severe AEs were observed up to week 16. Mild oral candidiasis was the most frequently reported AE (which has not been reported in our series), but none of the patients discontinued the treatment because of this. In the Italian registry, no clinical or serological evidence of viral reactivation was observed in their patients with concomitant viral hepatitis, consistent with data reported for other IL-17 inhibitors.

Finally, we would like to highlight the use of BMK in other forms of psoriasis not initially included in the technical data sheet, but for which there are no randomized clinical trials given its low incidence in the different forms of psoriasis which, given its efficacy, opens up a broad horizon for its treatment: acrodermatitis continua de Hallopeau [22], suberythrodermic psoriasis [23], or generalized pustular psoriasis [24].

One of the main limitations of our study is the sample size, but it allows us to obtain valuable information about the behavior of BMK in real clinical practice in terms of efficacy and safety. It would be interesting to delve deeper into the PROs (patient’s reported outcomes) beyond the evolution of the DLQI with the incorporation of adherence and satisfaction measures.

The main conclusions that can be drawn from the data presented are as follows:

- All patients showed rapid and sustained clearance of psoriasis with bimekizumab. BMK manages to achieve an optimal response in the main effectiveness variables measured: absolute PASI, BSA, DLQI, and VAS pruritus in 4 weeks, improving results in the short term to 16 weeks. Although rapid response time is not a quality that has been explored in many of the biologic treatments used to date in the management of moderate to severe psoriasis, BMK is a feature to be considered in cases where it is required.
- Bimekizumab was well tolerated. No side effects were found that have forced treatment suspension. Importantly, we did not find any of the class effects associated with IL17 inhibitor drugs (neutropenia, candidiasis, or inflammatory bowel disease) which have led to therapeutic discontinuation in numerous clinical trials or real clinical practice series with drugs in the same therapeutic class.
- Resolution of psoriasis was observed in all patients, irrespective of patient profile and psoriasis location. The therapeutic effect of bimekizumab (BMK) in the short term in areas that are difficult to treat is optimal and with significant levels of effectiveness.
- The efficacy of bimekizumab observed in our patients confirms that observed in clinical trials and in other clinical practice series published at the present time.
- The combination of bimekizumab’s efficacy, speed, durability, convenient dosing, and safety translates into improved patient outcomes.

## Figures and Tables

**Table 1:** Baseline characteristics of our patient sample (*n* = 22).

**Gender**, *n* (%)	
Male	10 (45.5%)
Female	12 (54.5%)
**Difficult-to-treat areas**, *n* (%)	
Ungual	10 (45.5%)
Scalp	16 (72.7%)
Palmoplantar	3 (13.6%)
Genital	9 (40.9%)
Inverse	12 (54.5%)
**Comorbidities**, *n* (%)	
Psoriatic arthropathy	3 (13.63%)
Diabetes mellitus	2 (9%)
Obesity	
Overweight (BMI 25–29.9)	9 (40.9%)
Grade I (IMC 30–34)	6 (27.3%)
Grade II (IMC 35–39.9)	1 (4.5%)
Grade III (IMC 40–49.9)	1 (4.5%)
Grade IV (≥ 50)	1 (4.5%)
Hypertension	6 (27.3%)
Dyslipidemia	5 (22.7%)
Depression	5 (22.7%)
Metabolic fatty liver disease	3 (13.6%)
**Latent tuberculosis infection**	3 (13.6%)
**Previous biological therapies**, *n* (%)	
Naïve	3 (13.6%)
1	8 (36.3%)
2	2 (9%)
3	4 (18.25)
5	3 (13.6%)
6	1 (4.5%)
8	1 (4.5%)
**Anthropometric variables**, $\bar{X}$ ± S	
Height (cm)	169.4 ± 7.2
Weight (Kg)	88.6 ± 25.8
BMI (Kg/m^2^)	26.09 ± 7.4
**Parameters of cutaneous involvement**, $\bar{X}$ ± S	
PASI baseline	14.2 ± 7.34
PASI w4	1.7 ± 3.49
PASI w16	0.3 ± 1.02
BSA baseline	18.95 ± 10.9
BSA w4	2.12 ± 5.2
BSA w16	0.43 ± 1.2
DLQI baseline	19.04 ± 5.7
DLQI w4	1.25 ± 2.1
DLQI sem 16	0.43 ± 1.3
EVAp baseline	7.33 ± 1.9
EVAp w4	0.68 ± 1.5
EVAp w16	0.43 ± 1.1

## Data Availability

The data presented in this study are available on request from the corresponding author.
